# The Role of Anesthesia during Intra-Arterial Mechanical Thrombectomy for theTreatment of Acute Ischemic Stroke

**DOI:** 10.7759/cureus.5831

**Published:** 2019-10-03

**Authors:** Joel Passer, Robert Maurer, Kadir Erkmen

**Affiliations:** 1 Neurosurgery, Temple University Hospital, Philadelphia, USA; 2 Neurosurgery, Penn State Milton S. Hershey Medical Center, Hershey, USA

**Keywords:** acute stroke, cerebral angiography, thrombectomy, general anesthesia, conscious sedation, cerebrovascular disease/stroke, ischemic stroke, cerebrovascular procedures

## Abstract

Background

Type of sedation (conscious sedation (CS) or general anesthesia (GA)) during Intra-arterial mechanical thrombectomy (IAMT) for treatment of acute ischemic stroke may affect patient outcomes. Previous studies suggested that CS cohorts have a higher probability of good outcome than GA cohorts. However, CS cohorts had lower initial NIH stroke scores (NIHSS). This study offers an investigation into outcomes after IAMT based on sedation type.

Methods

Patients at our institution who underwent IAMT for treatment of acute ischemic stroke caused by anterior circulation occlusion between 2013-2015 were included in the study. Primary endpoint was functional outcome on the modified Rankin Scale (mRS) at 90 days post-IAMT. Secondary endpoints included NIHSS at 48 hours post-IAMT, time from CT scan to puncture and from puncture to initial recanalization, recanalization as defined by the Thrombolysis in Cerebral Ischemia (TICI) score, intensive care and hospital length of stay, and all-cause in-hospital mortality.

Results

Thirty nine patients were included in analysis; 17 received GA and 22 received CS. Cohorts were similar in baseline characteristics, including NIHSS. The 90-day mRS was not significantly different between cohorts, as was the case for most secondary endpoints. Successful recanalization was higher in both groups than previously reported and a significantly higher TICI 3 recanalization rate was achieved in the GA cohort.

Conclusions

We show that equal outcomes are possible with either CS or GA if initial NIHSS is comparable. It seems reasonable for neuro-interventionalists to continue practicing using their personal preference for sedation. However, prospective randomized trials are still needed.

## Introduction

The treatment of acute ischemic stroke has significantly evolved over the last two decades due to the implementation of two major treatment modalities: Intravenous tissue plasminogen activator (tPA) therapy, which was shown to be effective in the mid-1990s, and more recently, intra-arterial therapies including intra-arterial tPA and mechanical thrombectomy devices [1]. While results of initial studies showed that intra-arterial therapy was not superior to standard of care, more recent large studies such as MR CLEAN, ESCAPE, and REVASCAT have shown intraarterial therapy to be safe and effective in the treatment of patients with acute ischemic stroke caused by proximal intracranial arterial occlusion in the anterior circulation [2-4]. With the publication of these studies, intra-arterial mechanical thrombectomy (IAMT) has become a widely used treatment for ischemic stroke. Although its utility has been well supported, the optimal protocol is still being explored [5,6].

Many factors likely affect patient outcomes. One such factor is the approach taken to patient sedation. Currently, the two most commonly used anesthetic options are complete general anesthesia (GA) or a more moderate level of conscious sedation (CS) [7]. Both routes have theoretical advantages and disadvantages. GA is thought to allow superior recanalization through immobilization of the patient, reducing risk of image degradation and reducing the risk of potential wire or catheter-induced vessel damage [8]. However, CS may allow for more timely recanalization as there is no procedural delay needed for arrival of the anesthesia team, induction of anesthesia and intubation among other factors. CS also allows for monitoring of neurological status and does not expose the patient to the inherent risks of general anesthesia, such as peri-procedural hypotension [8]. However, there is a risk that the patient under conscious sedation may acutely worsen for any number of medical reasons, requiring emergent intubation. If emergent intubation is necessary, patient outcomes are likely to worsen, due to delay while waiting for the anesthesia team to arrive, risk of aspiration and risk of death [9]. Some studies have observed a benefit to CS regarding mortality and long-term neurological outcomes, but opinions remain divided [10-12]. Furthermore, variability in workflow among institutions can confound the picture with some institutions being better equipped to utilize CS or GA for intervention for acute stroke.

Previous studies have shown that CS cohorts have a higher probability of good clinical outcomes, higher rates of recanalization, and faster treatment times than GA cohorts [8,10,13,14]. With this data, the American Heart Association, made new recommendations in 2015, stating that it might be reasonable to favor CS over GA during IAMT for acute ischemic stroke (Class IIb data, Level of Evidence C) [15]. However, in these same studies, the patients receiving CS had a lower initial NIHSS, which may have confounded the better outcomes. Furthermore, the data is older, ranging from 2002-2013. Lastly, rates of successful reperfusion (defined as TICI scores 2b or 3) were less than 50%. Advances in endovascular technology over the last few years, as well as more interventionalists performing higher volumes of procedures may reduce the benefit of CS observed in early studies.

In the current study, we present data from our own institution and seek to help build a consensus on this debated topic.

## Materials and methods

Study Design: 

This was a single center retrospective case-control study. The electronic medical records of patients included in the study were examined after proper approval from the institutional IRB and the relevant data was obtained and analyzed.

Subjects: 

All patients undergoing IAMT for acute ischemic stroke of the anterior circulation at Temple University Hospital between 01/2013 and 10/2015 were included in the preliminary subject list. Subjects were divided into two major groups: those who underwent intubation for the initiation of general anesthesia (GA), and those who were not intubated and received only mild sedation with a combination of low to moderate doses of midazolam, propofol and fentayl. Four neurointerventionalists performed procedures during this time period and choice of type of sedation was generally dictated by operator preference. Baseline demographics were collected including age, sex, location of vessel occlusion, initial NIH Stroke Scale score (NIHSS) and medical comorbidities.

Endpoints: 

The primary study endpoint was long-term functional outcome as assessed by the modified Rankin Scale (mRS) at 90 days post-IAMT. Our institution formally began documenting 90- day mRS scores in 2015, thus, for patients included in the study prior to 2015, mRS scores were estimated based on review of the electronic medical record. Secondary study endpoints included short term outcome as assessed by the NIHSS at 48 hours post-IAMT. It is standard protocol at our institution that all patients admitted with acute ischemic stroke undergo serial NIHSS assessments for at least 48 hours after admission and this variable was selected as a measurement of short-term response to IAMT. To assess the effect anesthesia may have had on delaying procedure onset or lengthening procedure duration, time from CT scan to groin puncture was calculated as well as time from groin puncture to initial recanalization. The technical success of the IAMT was determined by the degree of recanalization attained as defined by the TICI score. Further secondary endpoints included in-hospital mortality, intensive care length of stay, and total hospital length of stay.

Statistical Analysis: 

For continuous variables a Student’s T-test was performed to examine significance. For categorical values, a Chi-square test was performed. For binomial variables, a z-test of proportions was performed.

## Results

Patient Demographics: 

Forty patients were found to meet initial inclusion criteria in the study time period. One patient was excluded from the final analysis as the patient received neither GA nor CS. Of the thirty nine patients included in the final analysis, twenty-two received CS and seventeen underwent GA (Table 1). Three patients were converted from CS to GA during the procedure. Reasons for conversion included worsening mental status, vomiting while on the table, and combative behavior. One of the three patients had to undergo emergent cricothyroidotomy by general surgery during the procedure secondary to multiple failed attempts at intubation. These patients were included in the CS group for all analysis under the intent-to-treat principle. There were no differences in mean age or initial NIH Stroke Scale (NIHSS) between the two groups. In the CS group, twelve were female (60%) and the average age was sixty-two (62.05±4.14, Mean±SEM). The mean initial NIHSS was 17.30±1.30. In the GA group, eleven patients were female (58%) and the average age was sixty-seven (67.32±3.04). The mean initial NIHSS was 18.26±1.41. The two groups had no statistically significant differences with regards to the following acute ischemic stroke risk factors: hypertension, diabetes, history of atrial fibrillation and history of previous stroke. The location of the occlusion was similar between treatment groups.

**Table 1:** Baseline Patient Demographics SEM: Standard error of the mean, NIHSS: NIH Stroke Scale, ICA: Internal Carotid Artery.

	Conscious Sedation (n=22)	General Anesthesia (n=17)	P-value
Age	62.2±3.8 (mean + SEM)	67.8±3.3 (mean + SEM)	0.32
Gender	13 Female (59%)	10 Female (59%)	0.99
NIHSS at Presentation	16.7±1.4	19.1±1.2	0.62
Risk Factors			
Hypertension	15	13	0.57
Diabetes	5	8	0.11
Atrial Fibrillation	4	1	0.25
Previous Stroke	2	3	0.43
Location of Occlusion			
ICA	3	3	0.73
M1	13	9	0.70
M2	3	3	0.73
M3	1	0	0.37
Mixed	2	2	0.79

**Figure 1:**
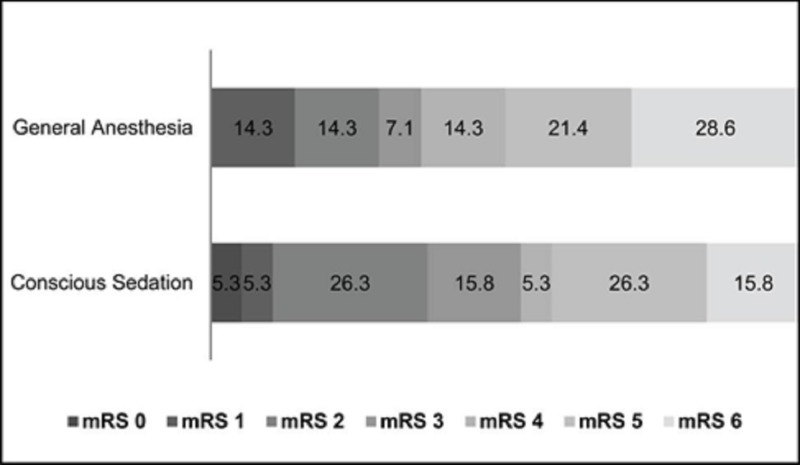
Outcome at 90 days, measured by modified Rankin Score Distribution of outcomes at 90 days on the modified Rankin Scale (mRS) in percentages in patients who received general anesthesia (n=17) or conscious sedation (n=22).

Primary Endpoint: 

The primary study endpoint was outcome as defined by mRS at 90 days (Figure 1). Three patients in the CS group and three patients in the GA group were lost to care following discharge. These patients were not included in the calculation of the primary endpoint but were included in the calculation of all other variables. Of the nineteen patients in the CS group who had adequate follow-up, seven were functionally independent at 90 days as defined by a mRS score of greater than 2. Four patients required low to moderate assistance with activities of daily living. Five patients were completely bedridden and dependent and three patients had expired by ninety days. Similarly, four of the fourteen patients with adequate follow-up in the GA group were functionally independent at ninety days. Three patients required low to moderate assistance and three patients were completely dependent. Four patients had expired by ninety days post-IAMT. A chi-square analysis of the mRS at 90 days revealed no difference (p=0.68). The proportion of patients functionally independent at 90 days, (29% [GA] vs. 36.9% [CS]) was not significantly different (p= 0.62). Furthermore, the proportion of patients with a poor outcome as defined by mRS ≥ 5, was also not significantly different (42.1% [CS] vs. 50% [GA], p= 0.65).

Secondary Endpoints:

Several secondary endpoints were examined (Table 2). Four patients in the CS group were not included in the calculation of NIHSS at 48 hours. Two of these four patients expired within 48 9 hours following IAMT. The other two patients either did not receive continued NIHSS assessment for 48 hours or documentation of the NIHSS was unable to be found in the medical record. One patient in GA group was not included in the NIHSS analysis for the same reason. The difference between the CS and GA groups was not observed to be significant (CS vs. GA: 16.7±1.6 vs. 18.9±2.0, p=0.32). All cause in-hospital mortality was observed in two patients in the CS group (9%) and two patients in the GA group (11%). One patient in the CS expired secondary to wire perforation during the procedure. The time from CT scan to puncture did not significantly differ between the groups (CS vs. GA: 1:39±0:13 vs. 1:30±0:6, p=0.62), nor did the time from puncture to recanalization (0:55±0:07 vs. 0:45±0:06, p=0.54). Hospital length of stay (LOS) (9.5±1.2 days vs. 10.3±1.4 days, p=0.4) and intensive care LOS (6.4±1.3 days vs. 7.3±1.2 days, p=0.74) were also not significantly different.

**Table 2:** Secondary Endpoints NIHSS: National Institute of Health Stroke Scale, ICU: Intensive Care Unit

	Conscious Sedation	General Anesthesia	P-Value
NIHSS at 48 Hours Post-op	16.7±1.6 (mean+SEM)	18.9±2.0 (mean +SEM)	0.32
30 day Mortality	2 Patients (9%)	2 Patients (11%)	0.79
90 day Mortality	3 Patients (15.8%)	4 Patients (28.6%)	0.42
Time from CT to Puncture (minutes)	99±13	90±6	0.62
Time from Puncture to initial recanalization (minutes)	55±7	45±6	0.54
Length of Stay (days)	9.5±1.2	10.3±1.4	0.40
ICU Length of Stay (days)	6.4±1.3	7.3±1.2	0.74

In determining the technical success of the thrombectomy, the degree of reperfusion was measured according to TICI score. In four patients under conscious sedation and three under general anesthesia, the catheter was unable to be advanced beyond the occlusion. In one patient under CS, a wire perforation occurred resulting in patient death. Successful recanalization as defined by TICI 2b/3 was achieved in fifteen patients (68%) in the CS cohort and fourteen patients (82%) in the GA cohort. In the CS group an incomplete degree of reperfusion was achieved in eleven patients and a complete reperfusion was achieved in five patients. In the GA group, incomplete reperfusion was achieved in three patients and thirteen patients had complete reperfusion following thrombectomy. A chi-square calculation was performed for the TICI scores in Table 3. Complete reperfusion was achieved in a significantly higher number of patients in the GA group (p=0.03). 

**Table 3:** Reperfusion success as assessed by TICI score TICI: Thrombolysis In Cerebral Infarction, CS: Conscious sedation, GA: General Anesthesia

	TICI 0,1	TICI 2a	TICI 2b	TICI 3	Total
CS	4	3	9	6	22
GA	3	0	2	12	17

## Discussion

The current debate about optimal anesthetic approach for patients undergoing IAMT is well founded. The arguments made in favor of both sides are logical and justified, however the evidence in the literature paints a murky picture of the most desirable protocol.

Previous studies have concluded CS to be as safe and effective as GA, with better patient outcomes. A meta-analysis of nine studies (published between 2010-2014) which compared results of type of sedation during IAMT, conducted by Brinjikji et al showed that GA was associated with lower odds of a favorable outcome and lower odds of successful recanalization, along with increased mortality [10]. There were no differences in time to groin puncture or time to recanalization in the meta-analysis. However, as reported in 6 of the 9 studies included in the meta-analysis, the GA cohort had a higher initial NIHSS, potentially confounding results. The lower recanalization rates in the GA cohort were thought to be secondary to presence of more challenging vascular occlusions and higher rates of intraprocedural hypotension in that group .

In our current study, we observed patients to have similar initial NIHSS in both groups. There was no significant difference observed in our primary endpoint of mRS at 90 days post-IAMT between patients who received CS and those who received GA. Similarly, there was no significant difference in 48 hour NIHSS, in-hospital mortality, LOS or ICU LOS. The GA cohort did not have a longer interval between CT scan and groin puncture. Furthermore, there was no significant difference seen between the two groups with respect to time needed to achieve initial recanalization. This is in-line with previous reports that have found the induction with general anesthesia did not delay procedure onset and did not prolong time to recanalization [10]. Brinjikji et al found time to groin was 136 minutes 20 seconds ± 54 minutes for general anesthesia compared with 117 minutes 20 seconds ± 56 minutes 20 seconds for conscious sedation (P = .24). Furthermore, mean time from symptom onset to revascularization was 329 minutes 43 seconds ± 173 minutes for general anesthesia compared with 354 minutes 51 seconds ± 265 minutes for conscious sedation (P = .17) [10].

We were able to achieve higher rates of TICI 2b/3 reperfusion in both groups than has been reported in the literature previously. The MR CLEAN group reported recanalization rates of less than 50% in both cohorts [14]. Our data shows TICI 2b/3 recanalization rates of 68% in the CS cohort (15/22) and 82% in the GA cohort (14/17). This may be a reflection of both improvements in technology and interventionalist experience and skill level compared to previous studies. Although both groups had a higher percentage of TICI 2b/3 scores than previously reported, we found that GA was associated with an even more superior level of recanalization as reflected by significantly higher TICI 3 scores in patients. This is not surprising given the theoretically better visualization achieved through GA attributable to reduced intra-procedural patient motion. Better imaging may reveal to the interventionalist an occlusion in a smaller, more distal vessel that would not otherwise have been seen had the patient received CS. Recognition of the smaller vessel occlusion would then lead to treatment of the occlusion and therefore a more satisfactory TICI result.

Although superior recanalization was achieved in patients under GA compared to CS, no clinical benefit was observed in these patients, as both short and long term assessment of neurological status were not significantly different between the groups. While the evidence supports a prognostic difference between partial recanalization (TICI 2a) and near complete recanalization (TICI 2b/3), there is currently some debate in the literature among the clinical significance and 12 difference in prognosis between TICI 2b and TICI 3 revascularization [16]. A recent study by Kleine et al. demonstrated that TICI 3 revascularization was associated with superior neurologic outcomes and shorter hospital stays when compared to TICI 2b but many authors have grouped TICI 2b and TICI 3 together for purposes of analysis [13]. In our current study, if TICI 2b and TICI 3 are grouped together, there was no significant difference in the quality of revascularization.

This study is limited by its retrospective nature and relatively small sample size. Differences in outcome within each cohort could be influenced by the specific drugs which were given to each patient during treatment, as suggested previously [17]. There may be a bias which is unaccounted for with regards to skill level and aggressiveness of the 4 neuro-interventionalists who treated patients in this study. It is possible that this could contribute to differences in TICI scores. Choice of type of sedation was left up to individual practitioner preference. There may be an additional selection bias here that cannot be accounted for. Regarding mRS estimation, it is unfortunate that our institution began recording formalized 90 day mRS outcome scores only relatively recently, as mRS estimation based on medical record review has been scrutinized [18].

Here, we show that with either method of sedation, we can achieve equal outcomes neurologically, without any delay in treatment and with improved revascularization rates. We have shown that the GA cohort did not experience any delays in time to initial recanalization versus the CS cohort. Both cohorts showed improved TICI 2b/3 outcomes compared to previous reports, with the GA cohort showing statistically significantly higher rates of TICI 3 outcomes. Our data shows no differences with regards to long-term neurologic status and mortality rates between the cohorts, differing from previous studies which give the advantage to CS cohorts [19-21]. It may be reasonable for individual neurointerventionalists to continue to practice based on their personal preference for patient sedation. Given that the current literature has failed to achieve a consensus, it may likely be that individual patients may derive benefit from different anesthetic approaches based on factors such as NIHSS at presentation, location of occlusion or risk factors for induction of general anesthesia. Further, large prospective randomized trials are needed to determine the optimal anesthetic approach and to determine if subsets of patients exist which may derive benefit from different approaches.

## Conclusions

We show that equal outcomes are possible with either CS or GA if initial NIHSS is comparable. It seems reasonable for neuro-interventionalists to continue practicing using their personal preference for sedation. However, individual patient factors are likely to result in patients being more suitable for either sedation or general anesthesia and clinicians should use their own judgment in appropriately selecting patients for one method or the other.
